# Supplementary material for: Integrating phylodynamics and historical records reveals decades-old introductions of PRRSV into Costa Rica via international swine trade
Source: bioRxiv. 2025 Aug 22:2025.08.22.671733. Preprint. [Version 1] doi: 10.1101/2025.08.22.671733 (PMC12393549; doi:10.1101/2025.08.22.671733)
Supplement: 1 [file NIHPP2025.08.22.671733V1-supplement-1.pdf]

bioRxiv preprint doi: https://doi.org/10.1101/2025.08.22.671733; this version posted August 22, 2025. The copyright holder for this preprint (which was not certified by peer review) is the author/funder. This article is a US Government work. It is not subject to copyright under 17 USC 105 and is also made available for use under a CC0 license.

Supplementary Material for:

Integrating phylodynamics and historical records reveals decades-old introductions of PRRSV into Costa Rica via international swine trade

Bernal León 1, Samiah Kanwar 2, Olga Aguilar 1, Idania Chacón 1, Gisela Cháves 1, David J. Spiro 3, Sana Tamim 4, Nidia S. Trovao 3

1 Virology Lab-LSE, Veterinary Services National Laboratories (LANASEVE), Animal Health National Service (SENASA), Ministry of Livestock and Agriculture (MAG), Heredia, Costa Rica

2 Department of Paediatrics and Child Health, The Aga Khan University, Stadium Road, Karachi, Pakistan

3 Fogarty International Center, National Institutes of Health, Bethesda, Maryland, 20892, USA

4 National Health Laboratory, National Institute of Health, Chak Shahzad, Islamabad, Pakistan

Corresponding author: Bernal León bernal.leon.r@senasa.go.cr

bioRxiv preprint doi: https://doi.org/10.1101/2025.08.22.671733; this version posted August 22, 2025. The copyright holder for this preprint (which was not certified by peer review) is the author/funder. This article is a US Government work. It is not subject to copyright under 17 USC 105 and is also made available for use under a CC0 license.

Supplementary Tables

Table S1. Sequences analyzed in this study.

GenBank ID	Collection year	Country
AB023782	1993	JPN
AB811787	2000	JPN
AF299407	1997	USA
AY585241	1997	KOR
DQ355796	1998	CHN
DQ475537	2001	USA
DQ477694	1999	USA
EU755455	2000	USA
EU755481	2001	USA
EU755538	2002	USA
EU755743	2004	USA
EU755777	2004	USA
EU755792	2004	USA
EU755884	1998	USA
EU755917	1999	USA
EU755939	1990	USA
EU755940	1990	USA
EU755992	2005	USA
EU756077	2002	USA
EU756201	2005	USA
EU756273	2005	USA
EU756349	2005	USA
EU756590	2006	USA
EU756674	2006	CAN
FJ899592	2003	CHN
JN651744	1990	USA

bioRxiv preprint doi: https://doi.org/10.1101/2025.08.22.671733; this version posted August 22, 2025. The copyright holder for this preprint (which was not certified by peer review) is the author/funder. This article is a US Government work. It is not subject to copyright under 17 USC 105 and is also made available for use under a CC0 license.

JN864948	2007	CHN
JQ087873	2010	USA
JX044140	1994	USA
JX138235	2007	KOR
JX857698	2011	CHN
KC469618	1995	USA
KC771287	2012	KOR
KC862575	2012	DNK
KC862576	1997	DNK
KC862577	2011	DNK
KC862578	2004	DNK
KC862580	2010	DNK
KC862584	2010	DNK
KF183946	2010	DNK
KF183947	2011	DNK
KF287141	2004	HKG
KF724413	2013	USA
KM386622	2014	KOR
KP256233	2014	KOR
KP998405	2005	TWN
KP998411	2011	TWN
KP998428	2002	TWN
KP998429	1992	TWN
KT257944	2014	USA
KU131559	2004	USA
KU318406	2015	USA
KX462792	2012	USA
KY348851	2000	USA
L40898	1991	CAN
MF663706	2016	USA
MH665696	2018	IND

bioRxiv preprint doi: https://doi.org/10.1101/2025.08.22.671733; this version posted August 22, 2025. The copyright holder for this preprint (which was not certified by peer review) is the author/funder. This article is a US Government work. It is not subject to copyright under 17 USC 105 and is also made available for use under a CC0 license.

MH665697	2018	IND
MK057529	2012	KOR
MK057530	2016	KOR
MK057532	2017	KOR
MK315208	2018	IND
MK315210	2018	IND
MK429987	2018	CHN
MK774669	2004	THA
MK774670	2012	THA
MN865569	2017	CAN
MW460545	2020	CHN
OL981187	2021	CHN
Z82995	1994	CAN
MW186707-CART3	2019	3CRC
MW186701-SRD8	2019	1CRC
MW186702-RA5PUSJ	2019	1CRC
MW186703-SRD7	2019	1CRC
MW186704-C4Pu	2019	1CRC
MW186705-RA7PUSJ	2019	1CRC
MW186706-R2SBHe	2019	4CRC
R4SBHe	2019	4CRC
511CA	2021	3CRC
460SACA	2021	3CRC

bioRxiv preprint doi: https://doi.org/10.1101/2025.08.22.671733; this version posted August 22, 2025. The copyright holder for this preprint (which was not certified by peer review) is the author/funder. This article is a US Government work. It is not subject to copyright under 17 USC 105 and is also made available for use under a CC0 license.

Table S2. Recombination events detected in the PRRSV-2 ORF5 sequences.

Sequence ID	Mayor parent	Minor parent	Beginning	Ending
JN651744_USA	KC771287_KOR	AB0237823_JPN	152	414
JX044140_USA	KC771287_KOR	AB023782_JPN	1176	414
MF663706_USA	Z82995_CAN	KF183947_DNK	710	309
MK315208_IND	MW4605450_CHN	KT257944_USA	917	126
MK315210_IND	MW460545_CHN	KT257944_USA	958	183
OL9811871_CHN	MK774670_THA	MW460545_CHN	292	1163

Table S3. Lineage classification of the analyzed sequences based on the Nextclade software.

Sequence Name	Lineage
AB023782_1993_JPN	L4
AB811787_2000_JPN	L5A
AF299407_1997_USA	L5A
AY585241_1997_KOR	L5A
DQ355796_1998_CHN	L5A
DQ475537_2001_USA	L5A
DQ477694_1999_USA	L5A

bioRxiv preprint doi: https://doi.org/10.1101/2025.08.22.671733; this version posted August 22, 2025. The copyright holder for this preprint (which was not certified by peer review) is the author/funder. This article is a US Government work. It is not subject to copyright under 17 USC 105 and is also made available for use under a CC0 license.

EU755455_2000_USA	L5A
EU755481_2001_USA	L1E
EU755538_2002_USA	L1E
EU755743_2004_USA	L5A
EU755777_2004_USA	L5A
EU755792_2004_USA	L5A
EU755884_1998_USA	L5A
EU755917_1999_USA	L5A
EU755939_1990_USA	L5A
EU755940_1990_USA	L9E
EU755992_2005_USA	L5A
EU756077_2002_USA	L1E
EU756201_2005_USA	L5A
EU756273_2005_USA	L5A
EU756349_2005_USA	L5A
EU756590_2006_USA	L5A
EU756674_2006_CAN	L5A
FJ899592_2003_CHN	L5A
JN651744_1990_USA	L5B
JN864948_2007_CHN	L5A
JQ087873_2010_USA	L5A
JX044140_1994_USA	L5B
JX138235_2007_KOR	L5A

bioRxiv preprint doi: https://doi.org/10.1101/2025.08.22.671733; this version posted August 22, 2025. The copyright holder for this preprint (which was not certified by peer review) is the author/funder. This article is a US Government work. It is not subject to copyright under 17 USC 105 and is also made available for use under a CC0 license.

JX857698_2011_CHN	L5A
KC469618_1995_USA	L5A
KC771287_2012_KOR	L5A
KC862575_2012_DNK	L5A
KC862576_1997_DNK	L5A
KC862577_2011_DNK	L5A
KC862578_2004_DNK	L5A
KC862580_2010_DNK	L5A
KC862584_2010_DNK	L5A
KF183946_2010_DNK	L5A
KF183947_2011_DNK	L5A
KF287141_2004_HKG	L5A
KF724413_2013_USA	L5A
KM386622_2014_KOR	L5A
KP256233_2014_KOR	L5A
KP998405_2005_TWN	L5A
KP998411_2011_TWN	L5A
KP998428_2002_TWN	L5A
KP998429_1992_TWN	L5A
KT257944_2014_USA	L5A
KU131559_2004_USA	L5A
KU318406_2015_USA	L5A
KX462792_2012_USA	L5A

bioRxiv preprint doi: https://doi.org/10.1101/2025.08.22.671733; this version posted August 22, 2025. The copyright holder for this preprint (which was not certified by peer review) is the author/funder. This article is a US Government work. It is not subject to copyright under 17 USC 105 and is also made available for use under a CC0 license.

KY348851_2000_USA	L5A
L40898_1991_CAN	L1I
MF663706_2016_USA	L1C.3
MH665696_2018_IND	L8E
MH665697_2018_IND	L8E
MK057529_2012_KOR	L5A
MK057530_2016_KOR	L11
MK057532_2017_KOR	L3
MK315208_2018_IND	L8E
MK315210_2018_IND	L8E
MK429987_2018_CHN	L5A
MK774669_2004_THA	L11
MK774670_2012_THA	L8E
MN865569_2017_CAN	L5A
MW460545_2020_CHN	L4
OL981187_2021_CHN	L4
Z82995_1994_CAN	L1E
MW186707-CART3_2019_3CRC	L4
MW186701-SRD8_2019_1CRC	L5A
MW186702-RA5PUSJ_2019_1CRC	L5A
MW186703-SRD7_2019_1CRC	L5A
MW186704-C4Pu_2019_1CRC	L5A
MW186705-RA7PUSJ_2019_1CRC	L5A

bioRxiv preprint doi: https://doi.org/10.1101/2025.08.22.671733; this version posted August 22, 2025. The copyright holder for this preprint (which was not certified by peer review) is the author/funder. This article is a US Government work. It is not subject to copyright under 17 USC 105 and is also made available for use under a CC0 license.

MW186706-R2SBHe_2019_4CRC	L5A
R4SBHe_2019_4CRC	L5A
511CA_2021_3CRC	L1E
460SACA_2021_3CRC	L1E

bioRxiv preprint doi: https://doi.org/10.1101/2025.08.22.671733; this version posted August 22, 2025. The copyright holder for this preprint (which was not certified by peer review) is the author/funder. This article is a US Government work. It is not subject to copyright under 17 USC 105 and is also made available for use under a CC0 license.

Supplementary Figures

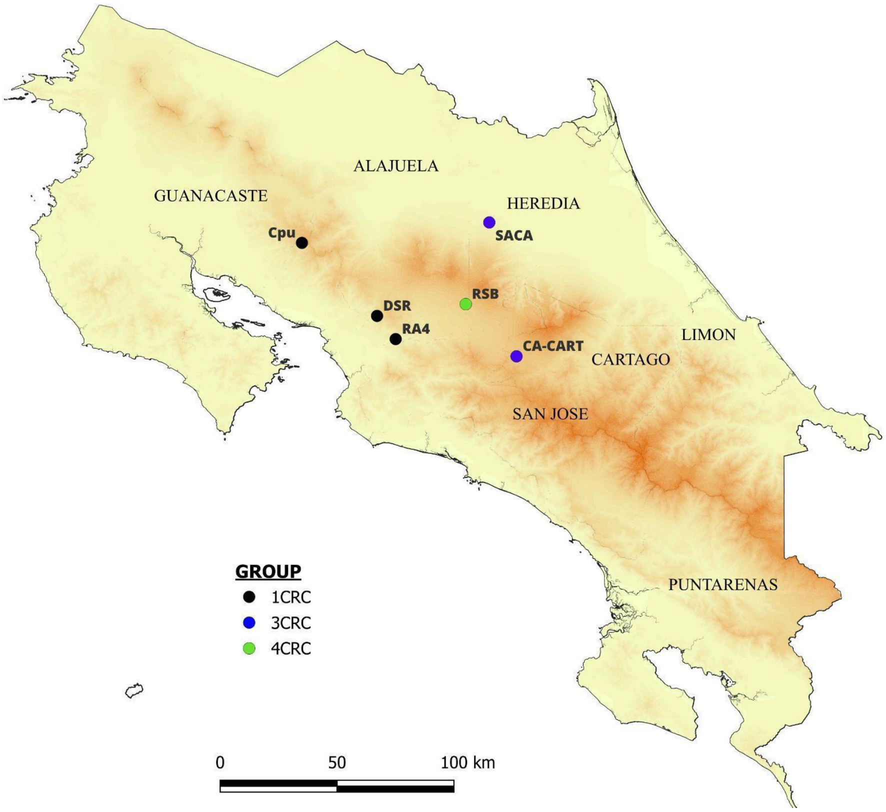

Figure S1. Geographical distribution of the Costa Rican PRRSV-2 clusters. The map indicates the farm locations from which samples were collected for this study. Each point is colored to correspond with the phylogenetic clusters defined in Figure 1: 1CRC (black), 3CRC (blue), and 4CRC (green). Farm codes are labeled for reference.

	L1	L1-3CRC	L3	L4	L5	L5-1CRC	L5-4CRC	L8	L9
**L1**	0.92	0.02	0.01	0.02	0.02	0.02	0.01	0.02	0.02
**L1-3CRC**	0.89	0.98	0	0.02	0.01	0.03	0.02	0.02	0
**L3**	0.87	0.84	N/A	0	0.01	0.01	0	0.01	0
**L4**	0.91	0.89	0.89	0.98	0	0	0	0.01	0
**L5**	0.89	0.87	0.9	0.93	0.97	0.01	0	0.01	0.01
**L5-1CRC**	0.85	0.85	0.84	0.88	0.89	0.98	0	0.02	0.01
**L5-4CRC**	0.86	0.85	0.85	0.89	0.9	0.87	1	0.01	0
**L8**	0.88	0.86	0.87	0.91	0.89	0.85	0.86	0.91	0.02
**L9**	0.9	0.86	0.88	0.93	0.92	0.87	0.87	0.9	N/A

bioRxiv preprint doi: https://doi.org/10.1101/2025.08.22.671733; this version posted August 22, 2025. The copyright holder for this preprint (which was not certified by peer review) is the author/funder. This article is a US Government work. It is not subject to copyright under 17 USC 105 and is also made available for use under a CC0 license.

Figure S2. Average pairwise genetic distance among lineages. Percent genetic difference within (intra-, highlighted in magenta) and between (inter-, highlighted in light blue) sublineages L1, L3, L4, L5, L8, and L9. The standard deviation of inter-lineage distances is highlighted in green.

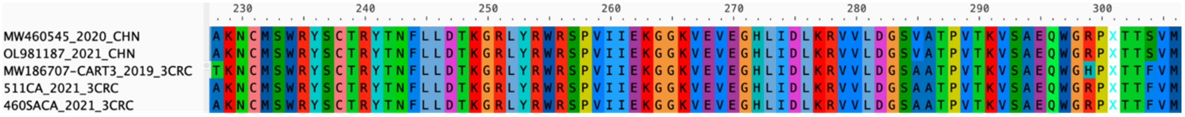

Figure S3. Amino acid comparison between lineages. Lineage 4 (L4) reference sequences from China and the Costa Rican sequences classified as lineage 1, sublineage E by Nextclade are shown, including the discordant sequence MW186707-CART3_2019_3CRC.
